# An Evaluation of Different 3D Cultivation Models on Expression Profiles of Human Periodontal Ligament Fibroblasts with Compressive Strain

**DOI:** 10.3390/ijms23042029

**Published:** 2022-02-12

**Authors:** Agnes Schröder, Ricarda Schöniger, Juliane Oeldemann, Gerrit Spanier, Peter Proff, Jonathan Jantsch, Christian Kirschneck, Niklas Ullrich

**Affiliations:** 1 Department of Orthodontics, University Medical Centre of Regensburg, 93053 Regensburg, Germany; agnes.schroeder@ukr.de (A.S.); ricarda.schoeniger@stud.uni-regensburg.de (R.S.); juliane.oeldemann@stud.uni-regensburg.de (J.O.); peter.proff@ukr.de (P.P.); 2 Department of Oral and Maxillofacial Surgery, University Medical Centre of Regensburg, 93053 Regensburg, Germany; gerrit.spanier@ukr.de; 3 Department of Medical Microbiology and Hygiene, University Medical Centre of Regensburg, 93053 Regensburg, Germany; jonathan.jantsch@ukr.de

**Keywords:** 3D cell culture, fibroblasts, PDLF, periodontal ligament, orthodontics, pressure, tooth movement

## Abstract

The effects of compressive strain during orthodontic treatment on gene expression profiles of periodontal ligament fibroblasts (PDLFs) have mostly been studied in 2D cell culture. However, cells behave differently in many aspects in 3D culture. Therefore, the effect of pressure application on PDLFs in different 3D structures was investigated. PDLFs were either conventionally seeded or embedded into different 3D structures (spheroids, Mebiol^®^ gel, 3D scaffolds) and exposed to compressive force or incubated without pressure. For one 3D scaffold (POR), we also tested the effect of different compressive forces and application times. Expression of an angiogenic gene (*VEGF*), a gene involved in extracellular matrix synthesis (*COL1A2*), inflammatory genes (*IL6*, *PTGS2*), and genes involved in bone remodelling (*OPG*, *RANKL*) were investigated by RT–qPCR. Depending on the used 3D cell culture model, we detected different effects of compressive strain on expression profiles of PDLFs. *COL1A2* was downregulated in all investigated 3D culture models. Angiogenetic and proinflammatory genes were regulated differentially between models. In 3D scaffolds, regulation of bone-remodelling genes upon compressive force was contrary to that observed in 3D gels. 3D cell culture models provide better approximations to in vivo physiology, compared with conventional 2D models. However, it is crucial which 3D structures are used, as these showed diverse effects on the expression profiles of PDLFs during mechanical strain.

## 1. Introduction

The dental discipline of orthodontics deals with the diagnosis, prevention, and treatment of malocclusions, which are of aberrant jaw and tooth positions in relation to the skull and the jawbone. The aim of orthodontic treatment is to create a stable and functional occlusion for the patient [1]. According to epidemiological studies, at least 70% of the population are affected by some form of malocclusion [2], which can be associated with a variety of complaints and secondary conditions such as psychological distress and functional discomfort in chewing, swallowing, and speaking [1]. Affected patients have an increased susceptibility to dental trauma [3], and misaligned teeth can impair oral hygiene favouring carious lesions [4] and, in the long term, periodontitis [5]. A pathological inflammation of the periodontium can lead to tooth loss in the long term and is associated with an increased risk of coronary heart disease [6]. For this reason, orthodontic treatment of malocclusions is considered to have an important preventive and curative effect. Due to its biomechanical properties, the periodontal ligament (PDL) connecting the tooth to its surrounding alveolar bone acts as a physiological mediator during orthodontic therapy and ensures that the effect of an orthodontic force is evenly distributed to the surrounding tissues [7]. Basically, orthodontic tooth movement is the result of pressure and tension zones created by force application within the periodontal ligament: New bone formation occurs at tension zones during orthodontic therapy, while resorption of the adjacent alveolar bone occurs at pressure zones of the PDL [8]. Of particular importance are periodontal ligament fibroblasts (PDLFs), which express more of the receptor activator of NF-kB ligand (*RANKL*) upon compression and, thus, increase the differentiation of osteoclasts, which are crucial for bone remodelling processes [9]. Healthy periodontal ligament consists of various cell populations such as endothelial cells, remnants of Malassez epithelial cells, immune cells, and cells associated with the sensory system, as well as bone and cementum-forming cells [10]. However, by far, the most abundant and functionally important cell type of periodontal connective tissue is the fibroblast [11]. This cell type plays a central role in the maintenance of physiological functions of the periodontal ligament [12], but also in the repair and regeneration processes of periodontal structures [13]. When an orthodontic force is applied to the PDL, there is an increased local release of neurotransmitters, cytokines, or even growth factors [9,14].The expression of inflammatory, vascular, and bone-modulating mediators triggers remodelling processes in the periodontal ligament and the surrounding alveolar bone [15]. PDLFs respond to compression with a markedly increased secretion of prostaglandin-endoperoxide synthase-2 (*PTGS2*) and prostaglandin-E2. This leads to increased expression of *RANKL*, which induces and increases osteoclastogenesis [9]. The increased activity of osteoclasts [1] leads to increased bone resorption at the pressure side [16], which ultimately enables tooth movement towards the compressed areas [17]. On the traction side, on the other hand, stretching of the periodontal fibre bundles provides certain stimuli that lead to the formation of new bone [17]. Our current understanding of many cellular processes is largely based on studies of homogeneously growing populations of cell cultures cultivated linearly on plastic surfaces [18]. This two-dimensional cell culture system is considered the gold standard for studying all cell types [19]. Advances in tissue engineering now enable three-dimensional cultivation and, thus, the mimicking of more realistic biochemical and biomechanical microenvironments for the cells under investigation [20]. These cultivation models ultimately approximate the in vivo situation of the human organism much better than it is the case with 2D cell cultures [21]. It is interesting to note that the gene expression of cells behaves differently in three-dimensional cultivation than in two-dimensional cultivation [21,22]. In their studies, Brezulier et al. (2020) were also able to demonstrate that PDLFs grown as spheroid culture in three dimensions show different reactions to mechanical strain than the same cell type after two-dimensional cultivation [23]. Thus, the question arises, to which extent the knowledge gained so far on 2D models regarding the response of PDLFs to mechanical forces in orthodontics corresponds to the physiology of the human organism and the actual response of PDLFs in the local tissue. Although the effect of three-dimensional cultivation has already been investigated, this study is the first to show a comparison of the expression patterns of PDLFs with a focus on OTM in different three-dimensional cell cultures under mechanical stress. The aim of this study is to identify the most suitable 3D culture model for future experiments on mechanically strained PDLF.

## 2. Results

### 2.1. Impact of Pressure Application on PDLFs Cultivated as Monolayers in Hydrogel, as Spheroids, or as Spheroids Embedded in Hydrogel

**Figure 1:**
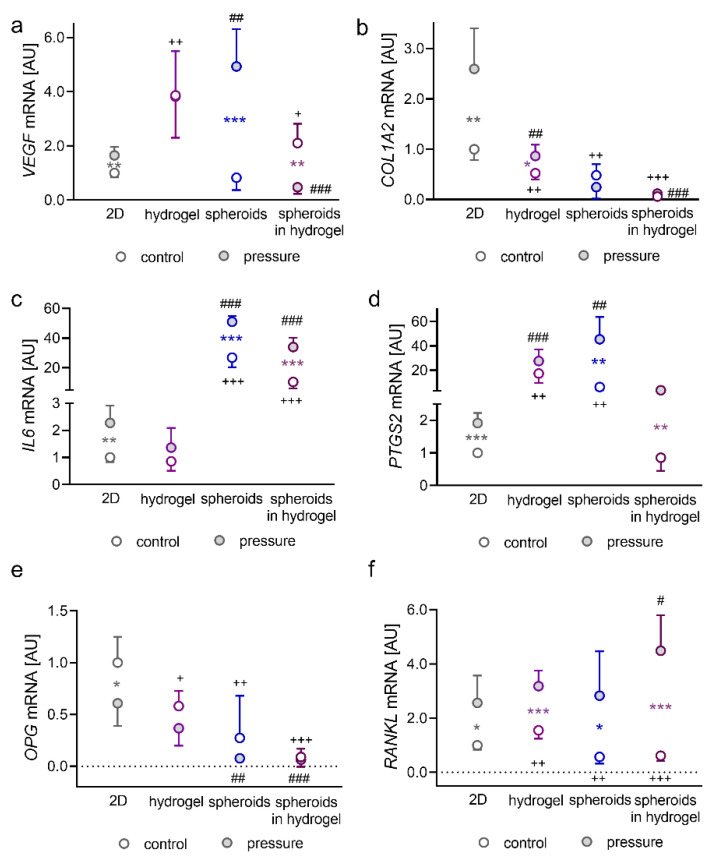
Impact of cultivation in hydrogels on the expression profiles of PDLFs cultured as monolayers or spheroids on the gene expression of *VEGF* (**a**), *COL1A2* (**b**), *IL6* (**c**), *PTGS2* (**d**), *OPG* (**e**), and *RANKL* (**f**); *n* = 9; Symbols represent mean values and the vertical lines show the standard deviation. *Statistics:* Welch-corrected ANOVA with Games–Howell multiple comparison tests. * pressure effect: * *p* < 0.05, ** *p* < 0.01, *** *p* < 0.001; ^+^ cultivation effect without compression: ^+^ *p* < 0.05, ^++^ *p* < 0.01, ^+++^ *p* < 0.001; ^#^ cultivation effect in combination with compression: ^##^ *p* < 0.01, ^###^ *p* < 0.001.

First, we determined the expression of the angiogenetic gene vascular endothelial growth factor (*VEGF*, Figure 1a,b). A significant increase in *VEGF* gene expression in standard 2D cell culture was detected after the application of compressive strain (*p* = 0.003; Figure 1a). Cultivation of single cells in hydrogels resulted in increased *VEGF* gene expression (*p* = 0.008), without further effect by pressure application (*p* > 0.999).

*VEGF* mRNA quantity increased with compressive strain in PDLFs formed to spheroids (*p* < 0.001; Figure 1a). However, embedded-in-hydrogel PDLF spheroids showed a downregulation of *VEGF* expression under pressure (*p* = 0.001; Figure 1a). Collagen-1-alpha-2 (*COL1A2*) encodes for a subunit of collagen fibrils and is, therefore, essential for extracellular matrix remodelling. *COL1A2* gene expression by PDLFs was upregulated by compressive strain in 2D culture (*p* = 0.004) and when embedded in hydrogel (*p* = 0.026; Figure 1c). This pressure effect disappeared when PDLFs were formed to spheroids (*p* = 0.368 and spheroids in hydrogel: *p* = 0.972). Cultivation of PDLFs in hydrogel reduced *COL1A2* mRNA expression without (*p* = 0.001) and with compressive strain (*p* = 0.002). This was also the case in the spheroid group (control: *p* = 0.002; pressure: *p* < 0.001) and the spheroids-in-hydrogel group (control: *p* < 0.001; pressure: *p* < 0.001; Figure 1b). During orthodontic tooth movement, a pseudoinflammation occurs with increased release and expression of inflammatory factors. Therefore, we tested the effect of different cultivation types on the gene expression of interleukin-6 (*IL6*) and prostaglandin-endoperoxide synthase-2 (*PTGS2*; Figure 1c,d). Compressive strain increased *IL6* mRNA expression in 2D culture (*p* = 0.018), in spheroids and in spheroids embedded in hydrogel (*p* < 0.001) but not in single cells in hydrogel (*p* = 0.578; Figure 1c). Notably, spheroid formation massively increased *IL6* gene expression without and with pressure application (*p* < 0.001; Figure 1c). Next to *IL6* mRNA, *PTGS2* gene expression was elevated with compressive strain in 2D culture (*p* < 0.001), in spheroids (*p* = 0.003) and in spheroids in hydrogel (*p* = 0.003; Figure 1d). We detected a significant increase in *PTGS2* mRNA in hydrogel (control: *p* = 0.003; pressure: *p* < 0.001) and after spheroids were formed (control: *p* = 0.005; pressure: *p* = 0.001). This effect was truncated when spheroids were embedded in hydrogel (control: *p* = 0.953; pressure: *p* = 0.066; Figure 1d). Lastly, we investigated the expression of genes involved in bone remodelling. Osteoprotegerin (*OPG*) gene expression was only reduced in 2D culture by compressive strain (*p* = 0.043; Figure 1e). However, overall *OPG* mRNA expression quantity was lower in all tested 3D cultures without (hydrogel: *p* = 0.013, spheroids: *p* = 0.009; spheroids in hydrogel: *p* < 0.001) and with compressive strain (hydrogel: *p* = 0.222, spheroids: *p* = 0.001; spheroids in hydrogel: *p* < 0.001; Figure 1e). Gene expression of receptor activator of NF-kB ligand (*RANKL*) was upregulated in all investigated setups (2D: *p* = 0.020, hydrogel: *p* < 0.001, spheroids: *p* = 0.040; spheroids in hydrogel: *p* < 0.001; Figure 1f). *RANKL* was increased under control conditions in hydrogel (*p* = 0.008), whereas it was reduced in spheroids (*p* = 0.013; spheroids in hydrogel: *p* = 0.005). Embedding spheroids in hydrogel even increased *RANKL* gene expression with compressive strain (*p* = 0.050; Figure 1f). Pressure application significantly elevated lactatdehydrogenase (LDH) release by PDLFs under compression in all cultivation models. There was no significant difference in LDH expression levels between all forms of 3D cell culture and 2D cell culture (Supplementary Figure S1a).

### 2.2. Impact of Pressure Application on PDLFs Cultivated in Different Solid 3D Scaffolds

**Figure 2:**
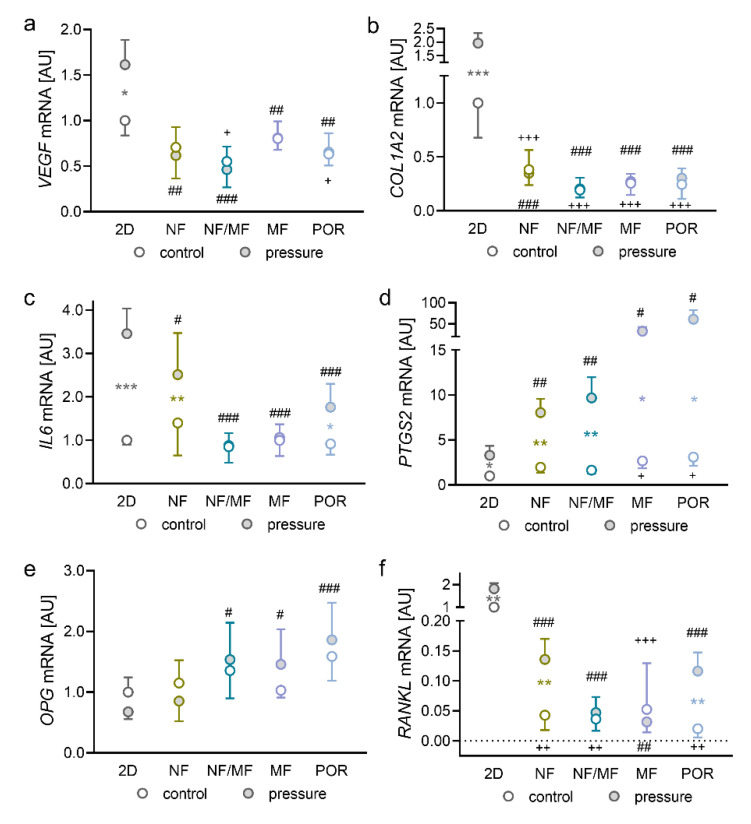
Impact of cultivation in different 3D scaffolds on the gene expression of *VEGF* (**a**), *COL1A2* (**b**), *IL6* (**c**), *PTGS2* (**d**), *OPG* (**e**), and *RANKL* (**f**); *n* = 6; symbols represent mean values and the vertical lines show the standard deviation. *Statistics:* ordinary ANOVA with Holm–Sidak’s multiple comparison tests (*COL1A2, IL6, OPG*) or Welch-corrected ANOVA with Games–Howell multiple comparison tests (*VEGF*, *PTGS2, RANKL*). * pressure effect: * *p* < 0.05, ** *p* < 0.01, *** *p* < 0.001; ^+^ cultivation effect without compression: ^+^ *p* < 0.05, ^++^ *p* < 0.01, ^+++^ *p* < 0.001; ^#^ cultivation effect in combination with compression: ^#^ *p* < 0.05, ^##^ *p* < 0.01, ^###^ *p* < 0.001.

Next, it was our goal to determine the possible effects of different solid 3D scaffolds on the expression profiles of PDLFs without and with compressive force application. Expression of the angiogenic gene *VEGF* was elevated in conventional 2D cell culture after compressive strain (*p* = 0.025; Figure 2a).

In contrast, the compressive strain showed no effect on *VEGF* expression in all of the investigated solid 3D scaffolds (*p* > 0.99). *COL1A2* gene expression was upregulated with compressive strain in the 2D model (*p* < 0.001; Figure 2b). Cultivation of PDLFs in 3D scaffolds reduced *COL1A2* gene expression in general without any effect by compression (*p* < 0.001; Figure 2b). The expression of the inflammatory gene *IL6* was elevated with compressive strain in 2D cell culture (*p* < 0.001), NF (*p* = 0.004), and POR scaffold (*p* = 0.040; Figure 2c). In the mixed NF/MF and the MF scaffold, no pressure effect on *IL6* gene expression was detectable (*p* = 0.997). In general, compressive induction of *IL6* was significantly reduced in the 3D scaffolds, compared with the 2D control (*p* < 0.001; Figure 2c). As expected, gene expression of *PTGS2* was upregulated in all tested cultivation models (2D: *p* = 0.033; NF: *p* = 0.001; NF/MF: *p* = 0.004; MF: *p* = 0.010; POR: *p* = 0.015; Figure 2d). Contrary to *IL6* gene expression, the pressure effect on *PTGS2* gene upregulation was even higher in the tested 3D scaffolds, compared with 2D cultivation (NF: *p* = 0.003; NF/MF: *p* = 0.004; MF: *p* = 0.011; POR: *p* = 0.015). Gene expression of the *RANKL* decoy receptor *OPG* was upregulated in the mixed NF/MF (*p* = 0.011), MF (*p* = 0.025), and POR scaffold (*p* < 0.001; Figure 2e) with compressive force. The amount of expressed *RANKL* was reduced in general in all 3D scaffolds examined, compared with the 2D control, regardless of the stimulation by pressure application (Figure 2f). A compressive upregulation of *RANKL* mRNA was significant in the 2D control (*p* = 0.007), NF (*p* = 0.009), and POR scaffold (*p* = 0.004; Figure 2f). Measurement of LDH in the supernatant of the samples revealed a significantly higher LDH release by PDLFs under compressive strain regardless of the type of cultivation with the exception of the POR scaffold, which induced a slightly higher LDH release already without pressure application, compared with the 2D control (Supplementary Figure S1b).

### 2.3. Impact of Different Magnitudes of Compressive Force on the Expression Profiles of PDLFs in the POR Scaffold

**Figure 3:**
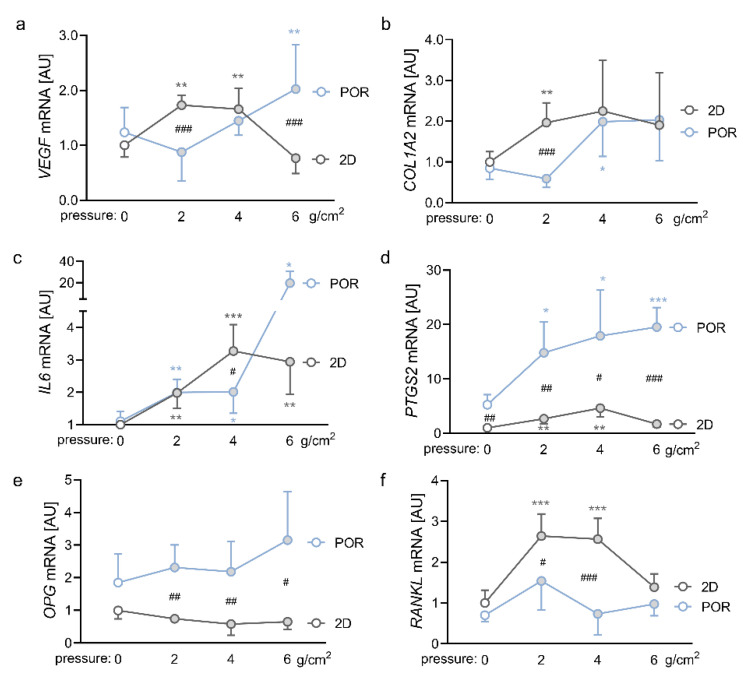
Impact of different compression forces on the gene expression of *VEGF* (**a**), *COL1A2* (**b**), *IL6* (**c**), *PTGS2* (**d**), *OPG* (**e**), and *RANKL* (**f**) in PDLFs cultured in the POR scaffold; *n* ≥ 7; symbols represent mean values and the vertical lines show the standard deviation. *Statistics:* Welch-corrected ANOVA with Games–Howell multiple comparison tests except for *VEGF*: ordinary ANOVA with Holm–Sidak´s multiple comparison tests. * pressure effect: * *p* < 0.05, ** *p* < 0.01, *** *p* < 0.001; ^#^ cultivation effect: ^#^ *p* < 0.05, ^##^ *p* < 0.01, ^###^ *p* < 0.001.

**Figure 4:**
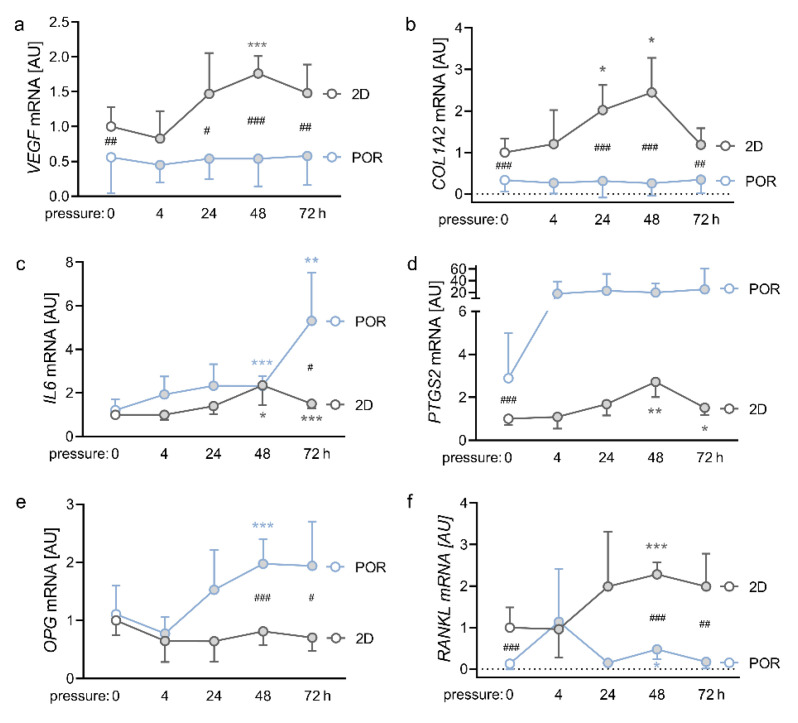
Impact of different compression times on the gene expression of *VEGF* (**a**), *COL1A2* (**b**), *IL6* (**c**), *PTGS2* (**d**), *OPG* (**e**), and *RANKL* (**f**) in PDLFs cultured in the POR scaffold; *n* ≥ 7; symbols represent mean values and the vertical lines show the standard deviation. *Statistics:* Welch-corrected ANOVA with Games–Howell multiple comparison tests. * pressure effect: * *p* < 0.05, ** *p* < 0.01, *** *p* < 0.001; ^#^ cultivation effect: ^#^ *p* < 0.05, ^##^ *p* < 0.01, ^###^ *p* < 0.001.

In this experiment, we compared the effects of different magnitudes of compressive force in the conventional 2D model and in the POR scaffold. To this end, we used ZnO_2_ plates, which applied 2 g/cm^2^, 4 g/cm^2^ or 6 g/cm^2^ on the PDLFs. In the conventional 2D model, compression with 2 g/cm^2^ (*p* = 0.003) and 4 g/cm^2^ (*p* = 0.008) increased *VEGF* gene expression significantly (Figure 3a). This effect was no longer detectable with a compressive force of 6 g/cm^2^ (*p* = 0.664). PDLFs cultured in the POR scaffold only showed an increase in *VEGF* gene expression after compression with 6 g/cm^2^ (*p* = 0.004) but not with 2 g/cm^2^ (*p* = 0.338) or 4 g/cm^2^ (*p* = 0.664), leading to a significant difference in *VEGF* gene expression between the 2D model and the POR scaffold with compressive forces of 2 g/cm^2^ and 6 g/cm^2^ (*p <* 0.001; Figure 3a). *COL1A2* gene expression was already increased at a force of 2 g/cm^2^ in 2D culture (*p* = 0.003), whereas significant upregulation of *COL1A2* in the POR scaffold was only visible at 4 g/cm^2^. (*p* = 0.047; Figure 3b). Expression of the inflammatory gene *IL6* was elevated in the 2D model with all tested compression forces (2 g/cm^2^: *p* = 0.003; 4 g/cm^2^: *p* < 0.001; 6 g/cm^2^: *p* = 0.005; Figure 3c). For PDLFs cultured in the POR scaffold, we observed a significant increase in *IL6* gene expression with all tested compression forces as well (2 g/cm^2^: *p* =0.002; 4 g/cm^2^: *p* = 0.045; 6 g/cm^2^: *p* = 0.043). A force of 6 g/cm^2^, however, boosted *IL6* gene expression even further (Abb. 4c). Pressure application elevated *PTGS2* gene expression in the 2D model and in the POR scaffold with 2 g/cm^2^ (2D: *p* = 0.006; POR: *p* = 0.012) and 4 g/cm^2^ (2D: *p* = 0.002; POR: *p* = 0.025; Figure 3d). A compression force of 6 g/cm^2^ increased *PTGS2* gene expression of PDLFs cultured in the POR scaffold even further (*p* < 0.001), whereas there was no significant compression effect at this force level in the 2D group (*p* = 0.087; Figure 3d). Notably, *PTGS2* gene expression was higher in all tested conditions in the POR scaffold, compared with the 2D model (0 g/cm^2^: *p* = 0.004; 2 g/cm^2^: *p* = 0.003; 4 g/cm^2^: *p* = 0.019; 6 g/cm^2^: *p* < 0.001). This was similar for *OPG* gene expression, where we detected increased expression after all tested pressure magnitudes in the POR scaffold, compared with the 2D model (2 g/cm^2^: *p* = 0.002; 4 g/cm^2^: *p* = 0.009; 6 g/cm^2^: *p* = 0.044; Figure 3e). In the conventional 2D model, gene expression of *RANKL* was elevated by compression with 2 g/cm^2^ and 4 g/cm^2^ (*p* < 0.001) but not with 6 g/cm^2^ (*p* = 0.238; Figure 3f). Cultivation of PDLFs in the POR scaffold abolished the pressure effect significantly (2 g/cm^2^: *p* = 0.033; 4 g/cm^2^: *p* < 0.001). Higher compressive forces resulted in higher LDH release, starting at a force of 2 g/cm^2^ under 2D conditions and 6 g/cm^2^ in the POR scaffolds (Supplementary Figure S1c). Therefore, a significant difference in LDH release between the 2D and POR-scaffold groups was only detectable without pressure application and with a force magnitude of 6 g/cm^2^.

### 2.4. Impact of Different Compression Times on the Expression Profiles of PDLFs in the POR Scaffold

Next, we compressed PDLFs cultivated in either conventional 2D culture or in the POR scaffold with 2g/cm^2^, for 4 h, 24 h, 48 h, or 72 h. *VEGF* gene expression showed a peak after 48 h of compressive strain in the 2D model (*p* < 0.001; Figure 4a), while PDLFs cultivated in the POR scaffold showed no significant compression effect on *VEGF* expression (*p* ≥ 0.99). Gene expression of *COL1A2* was elevated in the 2D model after 24 h and 48 h of compressive strain (24 h: *p* = 0.015; 48 h: *p* = 0.014; Figure 4b). Again, PDLFs did not increase *COL1A2* mRNA expression in reaction to compressive strain, when they were cultivated in the POR scaffold (*p* ≥ 0.999). Notably, gene expression of *COL1A2* was in general at a lower level, when PDLFs were cultured in the POR scaffold (Figure 4b). *IL6* gene expression was elevated in the 2D model only after 48 h of compression (48 h: *p* = 0.035; 72 h: *p* < 0.001; Figure 4c). The same effect was apparent in the POR scaffold (48 h: *p* < 0.001; 72 h: *p* = 0.010). After 72 h compression time, there was a significant increase in *IL6* gene expression in PDLFs cultivated in the POR scaffold, compared with PDLFs cultivated in the 2D monolayer (*p* = 0.016; Figure 4c). *PTGS2* gene expression was increased after 48 h of compression in the 2D model (48 h: *p* = 0.001; 72 h: *p* = 0.032; Figure 4d). In the POR scaffold, *PTGS2* gene expression was at a higher level, compared with the 2D model (*p* < 0.001; Figure 4d). This was also the case for *OPG* gene expression, which was upregulated in PDLFs cultivated in the POR scaffold after compressive strain (Figure 4e). As expected, *RANKL* gene expression was elevated after 48 h of compressive strain, when PDLFs were cultivated in the conventional 2D model (*p* = 0.004; Figure 4f). When cultivated in the POR scaffold, PDLFs showed reduced but significant compressive effects on *RANKL* upregulation after 48 h (*p* = 0.041). Contrary to *OPG* gene expression, *RANKL* gene expression was significantly lower in the POR scaffold, compared with the 2D model at baseline (0 h: *p* < 0.001) and after 48 h (*p* < 0.001) and 72 h of compression (*p* = 0.002; Figure 4f). From a compression period of 24 h and longer LDH levels increased in the 2D control, whereas PDLFs in the POR scaffold first showed reduced LDH values after 4 h, before LDH levels significantly increased as well after 24 h (Supplementary Figure S1d).

## 3. Discussion

In this study, we determined the expression profiles of PDLFs subjected to mechanical strain in different three-dimensional environments, also to ultimately identify the most suitable 3D environment for future in vitro 3D experiments with PDLFs under mechanical strain.

Thus far, it has been observed that the cell shape of adherent cells is completely altered in 2D culture compared with 3D culture media [24]. Furthermore, culturing cells under 2D conditions can result in a loss of cellular signalling pathways and changes in cell responses to certain stimuli [20,25,26]. Cells cultivated in 3D show a spatial distribution of cell-adhesion sites and a higher physical barrier of the surrounding matrix [27], so a three-dimensional matrix seems more accurate to correctly reproduce mechanical and biochemical cues in vitro [20,28]. The mechanical properties of the ECM hereby do not only affect the fibroblasts, but even more so, fibroblasts can also alter the mechanical properties of the ECM in an adequate three-dimensional environment [29]. Considering these aspects, three-dimensional cultivation seems crucial in the investigation of mechanotransduction in PDLFs.

To simulate compressive strain, ceramic discs with different weights were used. This uniform weight method is the most commonly used method for the investigation of orthodontic compressive strain on PDLFs [30]. As shown in a systematic review by Li et al., most studies used forces in the range between 1 g/cm^2^ and 4 g/cm^2^. Similar to our study, most studies in this review used a compressive force of 2 g/cm^2^ [30]. This differs from the proposed orthodontic forces by Schwarz (15–20 cN/cm^2^), and stress in the periodontal ligament can reach values up to 120 g/cm^2^ [30]. This is mainly due to the fact that we did not examine whole periodontal tissues with many cell lines in a complex extracellular matrix but a single cell line with the need for smaller forces. However, as PDLFs are embedded in a three-dimensional culture this time, it may be appropriate to increase disc weight, compared with 2D culture, as less compressive force may reach the individual cell in this experimental setup. For this reason, in addition to a 2 g/cm^2^ force, 4 g/cm^2^ and 6 g/cm^2^ forces were also examined on a POR scaffold in this study.

As of today, there are many different types of 3D culture. Mainly used 3D environments are naturally derived matrices, synthetic hydrogel-based scaffolds, solid porous scaffolds, and spheroids [24,31,32]. Naturally derived matrices, such as collagen, fibrin, or other complex decellularised matrices show on the one hand good biochemical properties, but are mostly limited in design flexibility and show batch-to-batch variability influencing reproducibility of experiments [27,32]. Synthetic porous scaffolds and hydrogels do not show these problems and can be altered in their biochemical and mechanical properties [24]. For this reason, we decided to use Mebiol^®^ Gel, a poly(ethylene glycol)-(PEG)-based hydrogel, and porous scaffolds on poly(ε-caprolactone) basis with different fibre morphologies. As a third option, we formed PDLFs to spheroids on low-adherent agarose. Compared with scaffold-based matrices such as hydrogels and solid scaffolds, the creation of spheroids is based on the basic principle of self-assembly, independent of biocompatible scaffold materials [20,33]. Thereby, the formation of the spheroid relies on intense cell–cell contacts and on the formation of their own extracellular matrix components [32,33,34]. In contrast to scaffold-based approaches, there is also a higher initial cell density [31] and a nutrient and waste gradient within the spheroid, resulting in high proliferative activity on the spheroid surface and necrotic cells in the core [20,33]. These morphologic aspects must be taken into account in the interpretation of the results. Finally, we embedded previously formed spheroids in Mebiol^®^ hydrogel to alter the mechanical properties of the ECM and to counter the typical low stiffness of spheroids, since the mechanical properties of the ECM can itself affect the gene expression of cell lines [20,31].

The vascular endothelial growth factor (*VEGF*) is involved in the neoformation and vasodilation of blood vessels [15]. This growth factor is targeted by the transcription factor HIF-1α, which is mainly induced by hypoxia but can also be mechanically stabilised [35,36,37]. Thus, *VEGF* is important in the reorganisation of the periodontal ligament during orthodontic tooth movement [38]. In general, a higher level of *VEGF* either after pressure application or under control conditions was higher in hydrogel and spheroids. PDLFs embedded in hydrogel showed higher *VEGF* expression values in general, with no effect by mechanical strain. Higher stiffness of the artificial hydrogel with the associated higher mechanical barrier of the ECM may be one reason for reduced mechanotransductive *VEGF* expression of PDLFs in Mebiol^®^ gel. The highest mechanotransductive induction of *VEGF* was seen in spheroids, followed by spheroids in hydrogels. The high *VEGF* induction by PDLFs formed to spheroids may be explained by the oxygen gradient present in spheroids [20,33]. Laschke et al. describe an upregulation of hypoxia-induced survival factors such as *VEGF* due to inefficient oxygen diffusion to cells in the core of spheroids [33]. In contrast, cultivation of PDLFs on solid 3D scaffolds showed in general lower levels of *VEGF* expression and no *VEGF* induction by pressure application, compared with 2D conditions. Better access to the surrounding medium due to an overall increase in surface area of the solid scaffold may have led to the better oxygen supply of the cells and, thus, to less *VEGF* expression. It was only from a force of 6 g/cm^2^ that more *VEGF* was expressed under pressure than in 2D. Since we suspect that the increased *VEGF* expression in hydrogels and spheroids is mainly generated by a lack of oxygen, solid 3D scaffolds might be better in this area to study the pure mechanical effect of OTM.

The gene collagen-type-1-alpha-2 (*COL1A2*) encodes the alpha-2 chain of collagen type I, the predominant collagen in the extracellular matrix of the periodontal ligament [39]. Upregulation of this gene, therefore, indicates higher collagen synthesis by PDLFs. The results show an overall clear drop in *COL1A2* expression during three-dimensional cultivation of PDLFs with spheroids embedded in hydrogel showing the lowest gene quantity. This may be due to matrix-dependent changes in cell morphology and a lower stimulus of an already existing 3D scaffold to produce ECM proteins [40,41]. In addition, it is described that the matrix-remodelling capacity of fibroblasts depends on the mechanical properties of the environment [29]. Thus, the altered mechanical properties of a three-dimensional matrix, compared with a 2D cell culture, may have reduced the remodelling activity of PDLFs. Flat-attached PDLFs on a rigid polystyrene base may undergo higher mechanical stress than in their spindle-shaped state in a 3D environment, even without additional application of pressure. This assumption is supported by the observation that PDLFs need higher mechanical strain in a 3D culture, compared with 2D conditions, to increase the expression of *COL1A2*. Berendsen et al., who exposed PDLFs in a collagen gel to tension, have made similar observations and only reported upregulated *COL1A1* expression levels under higher mechanical strain, while low forces showed no effect [42].

The secretion of proinflammatory cytokines is an integral step in the signalling cascade from external orthodontic force application to matrix remodelling, bone resorption, and finally tooth movement [8,9]. The investigated proinflammatory genes *IL6* and prostaglandin-endoperoxide synthase 2 (*PTGS2*) show different levels of expression highly dependent on the extracellular environment. It is remarkable that especially under spheroid formation the base level of inflammatory expression by PDLFs is massively increased, compared with PDLFs in 2D culture. A study by Vaheri et al. describes a new pathway of cell activation by PDLFs cultured in spheroids, leading to a massive proinflammatory, proteolytic, and growth factor response simultaneously undergoing a process characterised as programmed necrosis-like death [43]. This phenomenon was only observed with healthy adherent cells, while adherent cells derived from solid tumours form actively growing spheroids [43,44]. In contrast, PDLFs cultivated on solid 3D scaffolds showed a reduced base level of proinflammatory genes and a higher induction of *PTGS2* by pressure application, compared with the 2D culture. This lower level of proinflammatory gene expression by PDLFs on solid 3D scaffolds is in line with the findings by Htwe et al., who observed a reduced level of NF-κB activation by lung fibroblasts in a porous scaffold, compared with 2D, probably due to a lower TNF receptor expression pattern [45]. Subsequently, the reduced levels in NF-κB expression can downregulate the expression of proinflammatory genes [46]. In our opinion, the higher expression of proinflammatory cytokines without cell stimulation in hydrogels and spheroids does not approximate the in vivo situation, as a healthy periodontium without mechanical stimulation should have a low expression level of proinflammatory markers. From this point of view, three-dimensional cultivation on solid scaffolds seems to be more suitable for the investigation of orthodontic pressure application.

Receptor activator of NF-κB ligand (*RANKL*) and its decoy receptor osteoprotegerin (*OPG*) are the regulating cytokines in initiating bone resorption or apposition and, thus, have integral roles in the cellular processes of orthodontic tooth movement and tooth-root resorption [47,48]. *RANKL* triggers the differentiation of osteoclast precursor cells to osteoclasts and the activation of premature osteoclasts. As already shown in many experiments with PDLFs [49,50], in 2D on standard cell culture dishes a downregulation of *OPG* and simultaneous upregulation of *RANKL* under mechanical strain has been observed. Interestingly, in all investigated three-dimensional cell cultures, a downregulation of *OPG* under pressure application was no longer significant, while *RANKL* upregulation was seen in hydrogels, spheroids, spheroids in hydrogels, as well as on NF and POR scaffolds. With higher compressive forces and longer incubation time, *OPG* even seemed to be upregulated by tendency, while the magnitude of *RANKL* induction was reduced. A similar effect has been observed with osteoblasts sharing ultrastructural similarities with PDLFs [51], which expressed higher levels of *OPG* in correlation to higher magnitudes of compression, while *RANKL* mRNA expression reached its maximum at 2 g/cm^2^ and declined with higher mechanical forces [52]. This phenomenon was discussed by Shen et al. as a dual role of compressive stress with protective inhibition of osteoclastogenesis above a certain threshold [52]. This hypothesis is also supported by the observation that increasing the force magnitude does not accelerate tooth movement [53]. Additionally, the established 2D culture systems offered an easy-to-understand mechanical force induction, since one can assume that all adherent PDLFs flat-attached to the polystyrene base experience nearly the same force vector. In a complex 3D environment, such as the periodontal ligament in vivo, the initially applied force vector is probably divided and diverted into several smaller force vectors pointing into different directions. In some areas of the 3D matrix, this may even induce zones of tension favouring *OPG* over *RANKL* induction to initiate bone apposition [54,55]. This may be another explanation for the differences in *RANKL* and *OPG* expression in different 3D cultures and requires further research. Overall, with the exception of the NF/MF and MF scaffolds, all 3D cell culture models showed regulation of the *RANKL*/*OPG* system under pressure application, as it would be expected with respect to osteoclastogenesis in pressure zones of the periodontal ligament in vivo.

These results show that the choice of 3D culture significantly influences cell behaviour and should, therefore, be considered carefully. It must be taken into account that this study is limited in the number of genes investigated, which means that processes that we do not know about yet might be altered by one or the other form of 3D cell culture. It must also be stated that, as of today, there is a wide variety of three-dimensional matrices described in the literature, which we were not able to examine in total within the scope of this study, although we sought to investigate a broad spectrum of different forms of 3D cell culture. In addition, hydrogels and scaffold-based matrices, in particular, offer the possibility for modifications such as the adjustment of physical properties and coating with proteins, which provide almost infinite possibilities for optimisation and alteration of cell culture conditions [31]. This must be part of future investigations. Finally, it must also be mentioned that 3D cell cultures may be a good approximation to in vivo experiments, but animal experiments remain essential in further investigations of hypotheses based on in vitro experiments.

## 4. Materials and Methods

### 4.1. Isolation and Cultivation of PDLFs

Primary human PDLFs were isolated from the periodontal connective tissue of extracted human teeth of healthy donors that were free of decay and periodontal disease and were extracted for medical reasons. Tissue samples were cultivated in six-well cell culture plates (353046, Corning GmbH, Kaiserslautern, Germany) (37 °C, 5% CO_2_, 100% H_2_O) in complete media (DMEM High Glucose, D5671, Merck KGaA, Darmstadt, Germany) with 10% FBS (P30-3302, PAN-Biotech GmbH, Aidenbach, Germany); 1% antibiotic/antimycotic (A5955, Merck KGaA, Darmstadt, Germany); 1% L-glutamine (G7513, Merck KGaA, Darmstadt, Germany) and 100 µM ascorbic acid (A8960, Merck KGaA, Darmstadt, Germany) until proliferative outgrowth of adherently growing fibroblasts was observed. Cells were characterised by human PDLF-specific marker genes and a spindle-shaped morphology, as reported previously [49,56]. For the experiments, human PDLFs from the third to fifth passage were pooled from six patients (male: 3; female: 3; age: 17–27 years).

### 4.2. Cell Culture Experiments with Hydrogel and Spheroids

**Figure 5:**
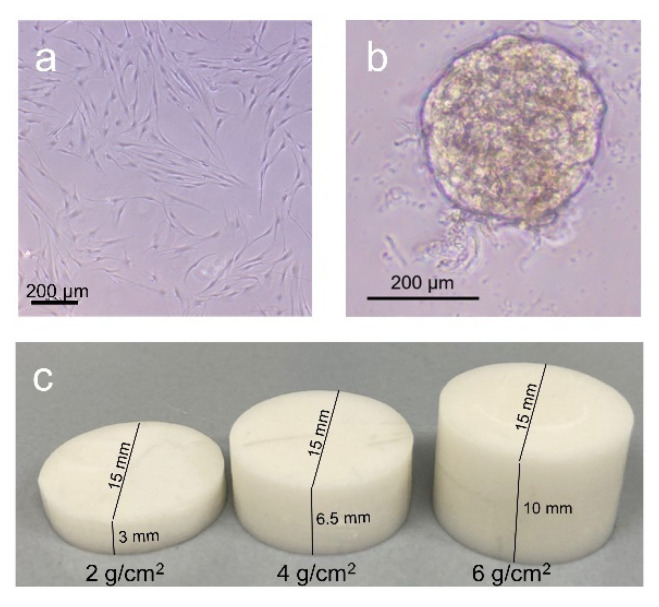
PDLFs either cultured as a conventional monolayer in Mebiol^®^ gel (**a**) or as spheroids in Mebiol^®^ gel (**b**) were compressed with ZnO_2_ plates of different heights (**c**) to achieve different compression magnitudes.

In the first cell culture experiment, we investigated the effect of compressive strain on the expression profiles of PDLFs cultivated in 2D on standard cell culture dishes or in 3D as monolayers in Mebiol^®^ gel (MBG-PMW20-5001, Cosmo Bio, Carlsbad, CA, USA; Figure 5a), as spheroid formation on standard cell culture dishes, or as spheroids in Mebiol^®^ gel (Figure 5b).

For spheroid formation, three sterile 96-well cell culture plates (353072, Corning GmbH, Kaiserslautern, Germany) were used per experiment. In each well, 75 µL 1% agarose (6351.5, Carl Roth GmbH, Karlsruhe, Germany) in PBS (14190-094, Thermo Fisher Scientific, Langenselbold, Germany) was added to prevent the PDLFs from growing on the bottom of the cell culture plate and to induce spherical agglomeration of the PDLFs (Figure 5b). After the agarose had been cured, 100,000 PDLFs per mL were seeded onto the three sterile 96-well cell culture plates and incubated for 72 h under cell culture conditions. Mebiol^®^ gel is a thermoreversible hydrogel that gels at 37 °C and changes to a liquid state at 4 °C. The Mebiol^®^ gel used (MBG-PMW20-5001, Cosmo Bio, Carlsbad, CA, USA) is available in freeze-dried form on the back of a T75 bottle (83.3911.002, Sarstedt, Nümbrecht, Germany). At least 72 h before seeding of the PDLFs or spheroids, 50 mL of DMEM High Glucose (D5671, Merck KGaA, Darmstadt, Germany) supplemented with 10% FBS (P30-3302, PAN-Biotech GmbH, Aidenbach, Germany), 1% antibiotic/antimycotic (A5955, Merck KGaA, Darmstadt, Germany), 1% L-glutamine (G7513, Merck KGaA, Darmstadt, Germany), and 100 µM ascorbic acid (A8960, Merck KGaA, Darmstadt, Germany) were added to the lyophilised Mebiol^®^ gel and initially stored at 4 °C for 3 h. The T75 bottle was then gently swirled and stored at 4 °C until use. A conventional 24-well cell culture plate (662-160, Greiner Bio-One GmbH, Frickenhausen, Germany) was used to implement the following experimental setup. Prior to seeding the PDLF single cells, PDLFs were trypsinised. For PDLFs in 2D culture, 50,000 cells per mL were dissolved in full medium, while 100,000 PDLFs were seeded in 200 µL Mebiol^®^ gel. For this purpose, the cell suspension was centrifuged at 800 rpm for 10 min. The cell pellet was taken up in Mebiol^®^ gel on ice to maintain the liquid state of the hydrogel. The spheroids that had been cultivated on the three 96-well cell culture plates were removed with a 1 mL pipette, transferred to two prepared 15 mL Falcon tubes (62.554.502, Sarstedt, Nümbrecht, Germany ), each containing 144 spheroids, and centrifuged at 1200 rpm for 15 min. One of the resulting cell pellets was dissolved in 3.1 mL of complete medium, while the other was taken up in 1.3 mL of ice-cold Mebiol^®^ gel. Prior to culturing the spheroids, 200 µL of 1% agarose in PBS were placed per well to prevent adherence of the PDLFs by reattachment to the plastic surface of the wells. After hardening of the agarose, 24 of the spheroids dissolved in the full medium could be placed in each well. For another group of experiments, 24 spheroids were seeded in 200 µL of Mebiol^®^ gel per well under refrigerated conditions. The cell culture plate was then removed from the ice to allow the Mebiol^®^ gel to solidify. After the complete hardening of the hydrogel, 300 µL of the full medium was added and finally incubated under cell culture conditions. After 24 h of preincubation, the corresponding pressure groups were loaded with sterile zirconium oxide (ZnO_2_) plates with a force of 2 g/cm^2^ (Cercon Base, 763,180, Dentsply Sirona GmbH, Bensheim, Germany; Figure 5c) for 48 h. These ceramic plates were milled in-house from zirconia blanks using the CAD/CAM process to the required dimensions and sterilised using hot air sterilisation at 200 °C for 2 h. After the incubation period, RNA was isolated and analysed with RT–qPCR. The supernatant was used for LDH assay (Supplemental Materials).

### 4.3. Cell Culture Experiments with 3D Scaffolds

To investigate the impact of different 3D scaffolds, we used the InoMATRIX morphology selection plate (811000-24, Biozym Scientific GmbH, Hessisch Oldendorf, Germany) in a 24-well plate format containing three-dimensional scaffold discs made of poly-ɛ-caprolactone (PCL) with a standard thickness of 200 µm per well. The fibre morphology of these scaffold discs varied per row of the 24-well plate. Thus, in the first row, there were six scaffolds of nanofibres (NFs) with a fibre diameter of 200 nm, while in the second row, there were six scaffolds of a mixture material of nanofibres and microfibres (NFs/MFs). Scaffolds solely based on microfibres (MFs) were placed in the third row with a fibre diameter of 5 µm. In the fourth row, there were scaffolds of porous fibres with a nanostructured surface (POR). For each series of experiments, a selection plate was used and the first row of a conventional polystyrene 24-well plate was used as a control. PDLFs were seeded in DMEM High Glucose (D5671, Merck KGaA, Darmstadt, Germany) supplemented with 10% FBS (P30-3302, PAN-Biotech GmbH, Aidenbach, Germany); 1% antibiotic/antimycotic (A5955, Merck KGaA, Darmstadt, Germany); 1% L-glutamine (G7513, Merck KGaA, Darmstadt, Germany), and 100 µM ascorbic acid (A8960, Merck KGaA, Darmstadt, Germany) and preincubated for 24 h under cell culture conditions. ZnO_2_ plates were then applied to half of the wells with a compressive force of 2 g/cm^2^ (Figure 5c) and for a period of 48 h. The other half remained untreated. Afterwards, the discs were removed, and RNA was isolated and analysed with RT–qPCR. The supernatant was used for the LDH assay (Supplemental Materials).

### 4.4. Cell Culture Experiments in POR Scaffolds with Different Magnitudes of Compression

To assess the optimal compression force in the POR scaffold, PDLFs were seeded in DMEM High Glucose (D5671, Merck KGaA, Darmstadt, Germany) supplemented with 10% FBS (P30-3302, PAN-Biotech GmbH, Aidenbach, Germany); 1% antibiotic/antimycotic (A5955, Merck KGaA, Darmstadt, Germany); 1% L-glutamine (G7513, Merck KGaA, Darmstadt, Germany), and 100 µM ascorbic acid (A8960, Merck KGaA, Darmstadt, Germany) either on conventional 24-well cell culture plates (662-160, Greiner Bio-One GmbH, Frickenhausen, Germany) or on 24-well INOMatrix plates (Porous POR PCL Morphology Single Type Plate, 811004-24, Biozym Scientific GmbH, Hessisch Oldendorf, Germany) and loaded with ZnO_2_ plates of different weights (2 g/cm^2^, 4 g/cm^2^, 6 g/cm^2^) (Figure 5c), for 48 h after 24 h of preincubation, under cell culture conditions. After the compression period, RNA was isolated and analysed with RT–qPCR. The supernatant was used for the LDH assay (Supplemental Materials).

### 4.5. Cell Culture Experiments in POR Scaffolds with Different Periods of Compression 

To assess the optimal compression time in the POR scaffold, PDLFs were cultured for 4, 24, 48, and 72 h either on conventional 24-well cell culture plates (662-160, Greiner Bio-One GmbH, Frickenhausen, Germany) or on 24-well INO matrix plates (Porous POR PCL Morphology Single Type Plate, 811004-24, Biozym Scientific GmbH, Hessisch Oldendorf, Germany) in DMEM High Glucose (D5671, Merck KGaA, Darmstadt, Germany) supplemented with 10% FBS (P30-3302, PAN-Biotech GmbH, Aidenbach, Germany); 1% antibiotic/antimycotic (A5955, Merck KGaA, Darmstadt, Germany); 1% L-glutamine (G7513, Merck KGaA, Darmstadt, Germany), and 100 µM ascorbic acid (A8960, Merck KGaA, Darmstadt, Germany). After 24 h of preincubation, the cells were either left untreated or compressed with ZnO_2_ plates (2 g/cm^2^). Depending on the periods to be investigated, the plates were left on the cells for 4, 24, 48, or 72 h. After the corresponding incubation time, RNA was isolated and analysed with RT–qPCR. The supernatant was used for the LDH assay (Supplemental Materials).

### 4.6. RNA Isolation

The Trizol method was used to isolate RNA. After incubation time, the supernatant was removed from the cells. To extract the RNA, 250 µL of phenol-containing RNA-Solv reagent (R6830-01, VWR International GmbH, Darmstadt, Germany) was pipetted per well. After a short reaction time, the entire volume was transferred to a previously prepared tube (Safe Seal, 72.706, Sarstedt, Nümbrecht, Germany) and 100 µL of chloroform (4432.1, Carl Roth GmbH, Karlsruhe, Germany) was added to each. Each sample was vortexed for 30 s, incubated on ice for 15 min, and centrifuged for another 15 min at 4 °C and 13,000 rpm (HERAEUS Fresko 17, Thermo Fisher Scientific Technologies, Langenselbold, Germany). The aqueous supernatant was transferred to a tube with 500 µL isopropanol (20.842.330, VWR International GmbH, Darmstadt, Germany). The samples were incubated at −80 °C overnight and then centrifuged for 30 min at 4 °C and 13,000 rpm, and the supernatant was removed. The RNA was further purified by adding 500 µL of 80% ethanol, and samples were centrifuged at 13,000 rpm and 4 °C for a further 10 min. After repeating the process, the RNA pellet was dried for at least 30 min. To determine the RNA concentration, the pellet of each sample was resuspended in 10 µL nuclease-free H_2_O_dd_ (T143.5, Carl Roth GmbH, Karlsruhe, Germany). Subsequently, 1.5 µL of each sample was measured in a photometer (NanoPhotometer N60, Implen GmbH, Munich, Germany).

### 4.7. cDNA Synthesis

Information about the expression profiles of the PDLFs to be investigated was obtained from the purified RNA. For this purpose, cDNA was synthesised from the isolated RNA using reverse transcriptase. In order to use the same RNA concentration for the reaction, the isolated RNA was diluted with nuclease-free H_2_O_dd_ (T143.5, Carl Roth GmbH, Karlsruhe, Germany) according to the measured concentration. Depending on the chosen approach, 4 or 8 µL RNA and 1 or 2 µL LunaScript RT SuperMix (E3010L, New England Biolabs GmbH, Frankfurt am Main, Germany) were added to the nuclease-free H_2_O_dd_. The samples were placed in a thermocycler (Thermocycler Tone 96G-Biometra, Analytik Jena GmbH, Jena, Germany) and incubated for 2 min at 25 °C and then 10 min at 55 °C. The reverse transcriptase was inactivated by heating to 95 °C for 1 min. The cDNA was then mixed with 45 µL nuclease-free H_2_O_dd_ (T143.5, Carl Roth GmbH, Karlsruhe, Germany) and stored at −20 °C until use.

### 4.8. Quantitative Real-Time PCR (RT–qPCR)

A primer mix consisting of 0.25 µL forward, 0.25 µL reverse primer (Table 1), 3 µL nuclease-free H_2_O_dd_ (T143.5, Carl Roth GmbH, Karlsruhe, Germany), and 5 µL Luna Universal qPCR Master Mix (M3003E, New England Biolabs GmbH, Frankfurt am Main, Germany) per sample was prepared for RT–qPCR. RT–qPCR was performed in duplicate for each sample. For this procedure, 1.5 µL of cDNA was pipetted onto a 96-well plate (712282, Biozym Scientific GmbH, Hessisch Oldendorf, Germany) and centrifuged. After the addition of 8.5 µL primer mix and repeated centrifugation, the plate was covered with an adhesive optical film (712350, Biozym Scientific GmbH, Hessisch Oldendorf, Germany). For evaluation of the expression profile, the plate was placed in the Realplex2 (Eppendorf Ag, Hamburg, Germany), and the appropriate programme (95 °C for 2 min and 45 cycles with 10 s 95 °C, 20 s 60 °C, 8 s 72 °C) was started. To determine the relative gene expression, the formula 2^−ΔCq^ (Livak et al., 2001), with ∆Cq = Cq (target gene) − Cq (geometric mean *PPIB/RPL22*) was used. Reference genes have been validated before [56]. For evaluation of the spheroid experiment, only reference gene *RPL22* was used, as *PPIB* showed reduced expression. All primers (Table 1) were designed according to the MIQE quality guidelines (Bustin et al., 2009).

### 4.9. Statistical Methods

All experiments were performed at least two times with three biological replicates each. RT–qPCR and LDH assays were performed as technical duplicates for each biological sample. *n* stated in the figure legends represents all biological replicates used for statistical analysis. Prior to statistical analysis, all absolute data values were divided by the arithmetic mean of the control group, to normalise data values to these controls. Quantitative data were presented as mean and standard deviation. We first determined the normal distribution of data using the Shapiro–Wilk test. For data with normal distribution, we performed ANOVA with Holm–Sidak’s multiple comparison tests, and for data that were not normally distributed, we performed Welch-corrected ANOVA with Games–Howell multiple comparison tests. Statistical analysis was performed with GraphPad Prism Version 9.1 (GraphPad Software, San Diego, CA, USA). Differences were deemed statistically significant at *p* < 0.05.

## 5. Conclusions

The expression of angiogenetic, proinflammatory, and osteogenic cytokines is vastly dependent on the environment in which the PDLFs are cultivated. Not only do cells, particularly extracellular-matrix-forming cells, behave very differently and probably more similar to in vivo conditions in a three-dimensional environment, compared with a standard 2D culture, as already stated in the literature [27,57,58], but variations among different types of 3D culture are also apparent. As Duval et al. have already stated, the gene expression of each 3D culture is specific in adaptation to its microenvironment [20]. Our initial results show higher levels of *VEGF* expression in hydrogels and spheroids, indicating reduced oxygen supply in these cultures. Furthermore, a higher expression level of proinflammatory cytokines in these three-dimensional cultures, even under control conditions without pressure application, seems to be less accurate in replicating the in vivo situation. Concerning osteoclastogenesis, RANKL was upregulated in all 3D cultures but not on NF/MF and MF scaffolds. Thus, as seen from current data, we recommend the usage of solid scaffolds in future research of pressure application on PDLFs, in particular the usage of scaffolds with a nanofibre (NF) or porous (POR) structure. On these scaffolds, we suggest a pressure application of 2 g/cm^2^ for 48 h as in 2D culture on standard cell culture dishes, as other force magnitudes and incubation times led to lower *RANKL* but higher *VEGF* expression.

## Figures and Tables

**Table 1:** Primer sequences for reference genes and target genes.

Gene Symbol	Gene Name	5′-Forward Primer-3′	5′-Reverse Primer-3′
*COL1A2*	Collagen-type-I-alpha-2	AGAAACACGTCTGGCTAGGAG	GCATGAAGGCAAGTTGGGTAG
*IL6*	Interleukin-6	TGGCAGAAAACAACCTGAACC	CCTCAAACTCCAAAAGACCAGTG
*OPG*	Osteoprotegerin	TGTCTTTGGTCTCCTGCTAACTC	ACGCTCCAGGACTTATACCG
*PPIB*	Peptidylprolyl isomerase A	TTCCATCGTGTAATCAAGGACTTC	GCTCACCGTAGATGCTCTTTC
*PTGS2*	Prostaglandin endoperoxide synthase 2	GAGCAGGCAGATGAAATACCAGTC	TGTCACCATAGAGTGCTTCCAAC
*RANKL*	Receptor activator of NFk-B ligand	ATACCCTGATGAAAGGAGGA	GGGGCTCAATCTATATCTCG
*RPL22*	Ribosomal protein L22	TGATTGCACCCACCCTGTAG	GGTTCCCAGCTTTTCCGTTC
*VEGF*	Vascular endothelial growth factor	TGCAGACCAAAGAAAGATAGAGC	ACGCTCCAGGACTTATACCG

## Data Availability

The data underlying this article will be shared at reasonable request to the corresponding author.

## Supplementary Materials

The following supporting information can be downloaded at: https://www.mdpi.com/article/10.3390/ijms23042029/s1.
