# Supplementary material for: An Evaluation of Different 3D Cultivation Models on Expression Profiles of Human Periodontal Ligament Fibroblasts with Compressive Strain
Source: Int J Mol Sci. 2022 Feb 12;23(4):2029. doi: 10.3390/ijms23042029 (PMC8876762; doi:10.3390/ijms23042029)
Supplement: Supplementary file 1 [file ijms-23-02029-s001.zip › ijms-1575941-supplementary.pdf]

International Journal of
Molecular Sciences

MDPI

Article

An Evaluation of Different 3D Cultivation Models on Expression Profiles of Human Periodontal Ligament Fibroblasts with Compressive Strain

Agnes Schröder [1], Ricarda Schöniger [1], Juliane Oeldemann [1], Gerrit Spanier [2], Peter Proff [1], Jonathan Jantsch [3], Christian Kirschneck [1,*,†] and Niklas Ullrich [1,*,†]

1 Department of Orthodontics, University Medical Centre of Regensburg, 93053 Regensburg, Germany; agnes.schroeder@ukr.de (A.S.); ricarda.schoeniger@stud.uni-regensburg.de (R.S.); juliane.oeldemann@stud.uni-regensburg.de (J.O.); peter.proff@ukr.de (P.P.)
2 Department of Oral and Maxillofacial Surgery, University Medical Centre of Regensburg, 93053 Regensburg, Germany; gerrit.spanier@ukr.de
3 Department of Medical Microbiology and Hygiene, University Medical Centre of Regensburg, 93053 Regensburg, Germany; jonathan.jantsch@ukr.de
* Correspondence: christian.kirschneck@ukr.de (C.K.); niklas.ullrich@ukr.de (N.U.)
† These authors contributed equally to this work.

Supplementary Materials

LDH Cytotoxicity Assay

Lactate dehydrogenase (LDH) assay was performed according to the manufacturer's instructions (04744926001, Roche), and absorbance was detected with an ELISA Reader (Multiscan GO, Thermo Fisher Scientific).

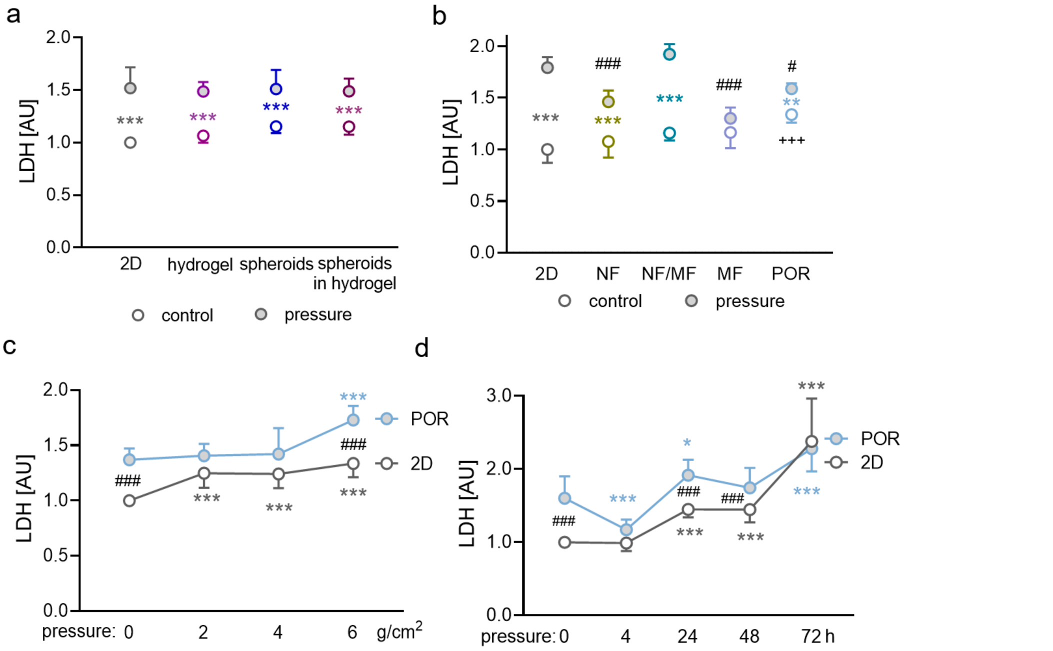

Figure S1. Impact of compressive strain on LDH release by PDLFs cultured in 2D, in hydrogel (**a**; n = 6) or different scaffolds (**b**; n = 6). LDH release without or with compression with different magnitudes of force (**c**; n = 12) and after different compression times in 2D or in the POR scaffold (**d**; n = 12); symbols represent mean values and the vertical lines show the standard deviation. *Statistics:* (**a**,**b**) Ordinary ANOVA with Holm–Sidak´s multiple comparison tests; (**c**,**d**) Welch-corrected ANOVA with Games–Howell multiple comparison tests. *pressure effect: * $p < 0.05$, ** $p < 0.01$, *** $p < 0.001$; +cultivation effect without compression: +++ $p < 0.001$; #cultivation effect in combination with compression: # $p < 0.05$, ### $p < 0.001$.
